# Predictive, preventive and personalised approach as a conceptual and technological innovation in primary and secondary care of inflammatory bowel disease benefiting affected individuals and populations

**DOI:** 10.1007/s13167-024-00351-x

**Published:** 2024-02-09

**Authors:** Laura Arosa, Miguel Camba-Gómez, Olga Golubnitschaja, Javier Conde-Aranda

**Affiliations:** 1 Molecular and Cellular Gastroenterology, Health Research Institute of Santiago de Compostela (IDIS), Laboratory 15, Trav. Choupana S/N, Building C, Level -2, 15706 Santiago de Compostela, Spain; 2 3P Medicine Research Unit, University Hospital, Rheinische Friedrich-Wilhelms Universität Bonn, 53127 Bonn, Germany

**Keywords:** Inflammatory bowel disease, Predictive preventive personalised medicine (PPPM / 3PM), Microbiota, Multi-level diagnostics, Biomarker panel, Individualised patient profile, Targeted treatments, Biologics, Small molecules, Primary and secondary care, Dietary and behavioural patterns, Mitochondria, Mitophagy, Health policy

## Abstract

Inflammatory bowel disease (IBD) is a global health burden which carries lifelong morbidity affecting all age groups in populations with the disease-specific peak of the age groups ranging between 15 and 35 years, which are of great economic importance for the society. An accelerating incidence of IBD is reported for newly industrialised countries, whereas stabilising incidence but increasing prevalence is typical for countries with a Westernised lifestyle, such as the European area and the USA. Although the aetiology of IBD is largely unknown, the interplay between the genetic, environmental, immunological, and microbial components is decisive for the disease manifestation, course, severity and individual outcomes. Contextually, the creation of an individualised patient profile is crucial for the cost-effective disease management in primary and secondary care of IBD. The proposed pathomechanisms include intestinal pathoflora and dysbiosis, chronic inflammation and mitochondrial impairments, amongst others, which collectively may reveal individual molecular signatures defining IBD subtypes and leading to clinical phenotypes, patient stratification and cost-effective protection against health-to-disease transition and treatments tailored to individualised patient profiles—all the pillars of an advanced 3PM approach. The paradigm change from reactive medical services to predictive diagnostics, cost-effective targeted prevention and treatments tailored to individualised patient profiles in overall IBD management holds a promise to meet patient needs in primary and secondary care, to increase the life-quality of affected individuals and to improve health economy in the area of IBD management. This article analyses current achievements and provides the roadmap for future developments in the area in the context of 3P medicine benefiting society at large.

## Preamble

### The gastrointestinal tract as the uniquely equipped system in the human body

The gastrointestinal tract (GIT) is a unique system in the human body which tolerates the luminal microbiota and protects involved tissues against harmful pathogenic flora and aggressive environmental agents. Thereby, the intestinal epithelium fulfils the barrier function and coordinates mucosal immunity intercommunicating microbiota of highly individual profiles and immune cells involved. This barrier is characterised by strict semi-permeability that allows for the absorption of nutrients and immune cell sensing but blocks potentially harmful invaders.

### Inflammatory bowel disease is a global health burden

Currently available statistics demonstrate inflammatory bowel disease (IBD) reaching the status of a global health burden which carries lifelong morbidity affecting all age groups in populations with the disease-specific peak age of onset ranging between 15 and 35 years. To this end, only about 10% of patients are diagnosed with IBD before reaching adulthood, which argues for the multi-factorial nature of the disease including the genetic component and environmental risk factors acting as the disease trigger. An accelerating incidence of IBD is reported for newly industrialised countries, whereas stabilising incidence but increasing prevalence is typical for countries with a Westernised lifestyle such as the European area and the USA [1].

### IBD as a chronic multifactorial immune-mediated inflammatory disease of the GIT with highly individual course and therapy outcomes

IBD is mainly comprised of Crohn’s disease (CD) and ulcerative colitis (UC) and is a group of immune-mediated disorders of the GIT, characterised by a chronic and recurring inflammatory response in the digestive tract mucosa. While UC is restricted to the colon and presents a continuous inflammation confined to the mucosal layer, CD shows a discontinuous and transmural inflammation that can occur anywhere in the GI tract. Although the aetiology of IBD is still largely unknown, the interplay between the genetic, environmental, immunological, and microbial components is decisive for the disease manifestation, course, severity and outcomes. Contextually, the creation of an individualised patient profile is crucial for cost-effective disease management in primary and secondary care. To this end, a key role of epigenetic regulation through environmental factors of risk has been postulated for the pathogenesis of IBD [1]. Factors such as cigarette smoking, sub-optimal dietary patterns, antibiotic and nonsteroidal anti-inflammatory drug (NSAID) use, limited or completely skipped breastfeeding, urbanisation and air pollution, amongst others, influence disease predisposition and severity at the individual level being also highly relevant for the entire population of corresponding regions. The proposed pathomechanisms include intestinal pathoflora and dysbiosis, chronic inflammation and mitochondrial impairments, amongst others, as discussed later on in this article, which collectively may reveal individual molecular signatures defining IBD subtypes, leading to clinical phenotypes, patient stratification and cost-effective protection against health-to-disease transition and treatments tailored to individualised patient profiles—the essential pillars of an advanced 3PM approach [2].

### Currently unmet patient needs, and the paradigm change from reactive medical services to 3PM approach

IBD is an incurable disease, and despite a spectrum of therapy options applied, a big portion of patients suffer from relapses and continuous inflammation leading to surgical removal of the intestine parts. The peak onset of the disease affects patients at a highly productive age. Typical IBD symptoms, including pain, diarrhoea or fever, interfere with patients’ daily tasks. Also, being affected by a lifelong disease can be an emotional burden. Hence, some IBD patients present with anxiety, anger or fear [3]. Furthermore, patients with UC have an increased incidence of other serious diseases such as colon cancer [4], making this disease to enormous socio-economic burden.

Currently, applied therapeutic approaches are based on biologics, immunomodulators, aminosalicylates, and corticosteroids [5]. Due to a highly heterogeneous patient cohort considering individual risk factors, pathophysiologic mechanisms, location of adverse health effects, disease initiation and progression, amongst others, therapeutic efficacy and outcomes are highly individual and any prognosis is rather vague challenging the treatment strategy. Contextually, 30% of patients are primary non-responders and 50% are secondary non-responders to anti-TNFα therapies which represents a major breakthrough in IBD treatments [6].

Overall, the paradigm change from reactive medical services to predictive diagnostics, cost-effective targeted prevention and treatments tailored to individualised patient profiles in overall IBD management holds a promise to meet patient needs in primary and secondary care, to increase the life quality of affected individuals and to improve health economy in the area. This article analyses current achievements and provides the roadmap for future developments in the area in the context of 3P medicine benefiting society at large.

## IBD treatments: individual IBD subtypes are in focus

The use of biologic agents has revolutionised the treatment of IBD. These drugs dramatically reduced the use of steroids, hospitalisations and the need for surgery. Despite the great success of these therapeutic approaches, many IBD patients do not respond adequately to these drugs. For instance, 10–30% of patients with IBD do not respond to anti-TNF therapy, and 20–40% lose effectiveness over time [7]. In the same way, the estimated vedolizumab (VDZ) [8, 9] and ustekinumab (UST) [10] primary response is around 60–70%, and recent reports suggest similar results for the small molecules [11, 12]. In addition, taking these immunomodulators can blunt physiological immune responses, leading to serious side effects such as infections. As a result, a considerable percentage of patients are treated with expensive compounds and exposed to undesirable side effects without obtaining any therapeutic benefit. Therefore, we cannot be content with the current IBD therapeutic armamentarium, and finding new classification criteria able to maximise therapeutic efficacy and minimise the probabilities of adverse events is still a challenge. This is even more relevant when new small molecules (upadacitinib, filgotinib and etrasimod) and monoclonal antibodies (risankizumab, mirikizumab or guselkumab) are/may be approved in the near future [13–15]. Definitively, having more therapeutic options will suppose an enormous advantage for the patients who are refractory to the existing therapies. However, gastroenterologists will also confront more complex decisions in prescribing those drugs to a particular patient. Moreover, this scenario of increasing available treatments, but lacking appropriate predictive/classification criteria, could expose IBD patients to multiple therapeutic failures with the concomitant risk of irreversible tissue damage because of uncontrolled inflammation. Therefore, the inclusion of routinely used predictive algorithms for each treatment, allowing clinicians to make informed decisions on the suitability of a given therapy to a particular patient, is of utmost importance.

## Clinical and patient-derived predictive data: focus on the tailored therapeutic algorithms

There is a multitude of studies trying to establish associations between patients’ parameters and their responsiveness to biologics. Age, gender, disease duration and biochemical and faecal markers have shown some degree of predictive power. However, the existence of inconsistencies and contradictory results limit their ability to take tailored therapeutic algorithms [16]. Therefore, we will focus on the most recent findings related to this issue (Table 1).

**Table 1:** Clinical and patient-derived predictive parameters and their association with the response to several biologic treatments. Focus on the tailored therapeutic algorithms. This table highlights the most recent factors, clinical parameters and clinical algorithms able to predict the response to the most prescribed biologic in IBD patients

	FACTOR	LEVEL	PREDICTION	*Ref*
Early parameters	Lichtiger index (LI)	Low	R to VDZ therapy in UC at 26–56 weeks post-initiated treatment	*19*
	Mayo score (PMS)	Low		20
	Mayo endoscopic subscore (MES)	Low		21
	Stool frequency, rectal bleeding, faecal calprotectin, faecal lactoferrin	Low in faeces	R to VDZ therapy in UC and CD patients	22, 23, 24
	Intestinal ultrasound criteria	Low	R in UC patients at 9 months post-initiated treatment	25
Blood and intestinal tissue biomarkers	TREM-1	Low in whole blood cells and intestinal mucosa	R to anti-TNF therapy in CD and UC patients	26
	OSM	High in the colon or serum at baseline	R to anti-TNF therapy in UC and CD patients	29, 30, 31
	IL-23	Low mucosal expression at baseline	NR to UST therapy	33
	Glycosylation IgG	Low levels in serum	R to IFX or ADA therapy in UC	34
	NAMPT	High levels in serum	NR to ADA therapy	35
	Vitamin D	Low levels in serum at baseline	NR to VDZ therapy in UC and CD patients	36, 37
	Neutrophil-to-lymphocyte ratio	Low in pheripheral blood at baseline	R to anti-TNF therapy in UC patients at 54 weeks post-initiated treatment	38
	Platelet-to-lymphocyte ratio			
	CD4^+^ α4β7^+^ T lymphocytes	High in pheripheral blood at baseline	R to VDZ therapy in UC patients at 14 weeks post-initiated treatment	39
	CD4^+^ α4β1^+^ T lymphocytes	High in pheripheral blood at baseline	R to VDZ therapy in CD patients at 14 weeks post-initiated treatment	
	Histologic analysis of ileal microvillar length	1.35 to 1.55 μm	R to UST and VDZ therapy in CD patients	40
Clinical data	PMS, C-reactive protein, cholesterol	AUROC at baseline	Early clinical response	41
	Malnutrition Universal Screening Tool (MUST) score	Increased at baseline	NR to VDZ therapy	42
	Clinical decision support tool (CDST)	CDST > 19 at baseline	R to VDZ therapy in CD patients	43

### Early parameters

Predictive markers measured at baseline would offer the opportunity to choose the most appropriate medication for a particular patient prior to its initiation. While this is an evident ideal situation, the detection of non-responders at initial stages of treatment should not be underestimated since early therapy discontinuation could save weeks of uncontrolled inflammation until an effective drug is applied.

It has been reported that clinical response at 6–8 weeks after induction therapy with golimumab or adalimumab (ADA) was a significant predictor of clinical remission at later time points (26 and 56 weeks) [17, 18]. In the same way, studies including different cohorts of patients revealed a strong association between the early clinical response, defined by the Lichtiger index (LI), partial Mayo score (PMS) and Mayo endoscopic subscore (MES) and long-term endoscopic remission in vedolizumab treated UC patients [19–21]. Although clinical and endoscopy assessments show promising results as therapeutic decision tools, the routine implementation of endoscopic evaluations after the start of medication is limited by the invasiveness nature of the technique and the high costs. Thereby not all health systems would be able to support that economic pressure, and probably, part of the general public would be excluded from that personalised approach. In that context, the detection of easy-to-read and well-defined markers of mucosal inflammation gains importance for their feasible monitoring at multiple time points since the administration of the drug. This is the case of patient outcomes such as stool frequency, rectal bleeding [22] or faecal calprotectin, which a reduction in its levels from the start of vedolizumab treatment is associated with the response to that therapy in patients with UC and CD [23], and something similar occurs with faecal lactoferrin [24], highlighting the potential of early follow-up of faecal biomarkers as predictors of endoscopic and histologic remission at later stages. In line with this, very recently, Allocca et al. suggested the implementation of intestinal ultrasound as a less invasive and more friendly method for more exhaustive patient assessment under biologics treatment. In a prospective observational study, these authors found that a significant improvement in the Milan ultrasound criteria (a validated score that correlates with endoscopic activity in UC patients) at 12 weeks post-initiation of treatment predicts endoscopic remission (assessed by colonoscopy and MES) at 9 months after starting therapy with several biologics [25]. Future validation in larger cohorts of patients will be necessary to adopt this technique on a regular basis. Nevertheless, this study builds the foundations for a novel non-invasive approach to closely monitor responsiveness to biologic therapy, allowing early decisions on drug continuation/discontinuation.

### Blood and intestinal tissue biomarkers

Single easy-to-read predictive markers are the goal that has been pursued in the last several years by the IBD research community. Although the clinical application of such a biomarker has not been yet achieved due to discrepancies amongst studies, several factors arose with the potential to discriminate responders from non-responders at baseline, contributing to targeted prevention, improving individual outcomes and decreasing therapy expenditures. For instance, low expression of the triggering receptor expressed on myeloid cells 1 (TREM-1) in whole blood cells and intestinal mucosa predicts future responders to anti-TNF therapy in CD and UC patients [26], although a meta-analysis of publicly available colon biopsies microarray datasets and confirmation experiments made in CD patients’ blood showed opposite results [27]. Differences in the composition of both cohorts and the assessment of clinical response might explain those discrepancies. Nevertheless, functional studies revealed that monocytes expressing high levels of TREM-1 failed to differentiate into anti-inflammatory macrophages under anti-TNF treatment [28], thereby showing a putative mechanism by which IBD patients with increased TREM-1 do not respond to TNF blockers.

Another appealing potential predictive biomarker for biologic therapy is oncostatin M (OSM). Increased OSM levels in the colon or serum at baseline predict clinical and mucosal remission in both UC and CD patients [29–31]. However, as opposed to that observed for TREM-1, the specificity of OSM to only anticipate anti-TNF response is more controversial since in CD subjects it is observed such effect [32], but when combined with CD and UC patients, OSM seems to act as a broader predictive marker for different monoclonal antibodies [30]. Further experiments designed to clarify those issues will be mandatory before clinicians can use OSM for decision-making.

TREM-1 and OSM are probably the best characterised and positioned to translate from bench to bedside. However, the list of potential soluble and tissue predictive factors is increasing, and other proteins capable of discriminating the therapeutic outcome from baseline are being identified. Low mucosal IL23 expression at baseline relates to UST treatment resistance [33]. Glycosylation experiments performed in the serum of patients with UC revealed that certain glycan structures exposed in the immunoglobulins might predict the response to infliximab (IFX) or ADA [34]. Similarly, the classification of IBD patients according to their basal circulating levels of nicotinamide phosphoribosyltransferase (NAMPT) discriminates with high sensitivity and specificity for the patients that will benefit from ADA treatment [35]. Noteworthy, a high proportion of the studies relate to anti-TNF response prediction. However, very recently, novel biomarkers for VDZ therapy response were isolated from IBD patients’ blood samples. This is the case with vitamin D and several extracellular matrix components, in which circulating levels might be useful to isolate patients in which VDZ therapy is most likely to fail [36, 37].

Apart from serum proteins, immune cell phenotyping in peripheral blood has also been potentially indicative of the therapeutic efficacy of several biologics. UC patients who achieved clinical and endoscopic remission at 54 weeks post-initiation anti-TNF therapy presented decreased neutrophil-to-lymphocyte and platelet-to-lymphocyte ratio at baseline [38]. Moreover, basal upregulated CD4^+^ α4β7^+^ T lymphocytes in UC patients was associated with VDZ response, while in CD patients, the subpopulation of T lymphocytes CD4^+^ α4β1^+^ is more indicative of VDZ therapy success at 14 weeks follow-up [39]. In line with this, histologic analysis of ileal microvillar length has been also found to be predictive of UST and VDZ response in CD patients [40]. Generally, these results highlight that combining conventional laboratory techniques with novel predictive parameters would make feasible the development of useful innovative screening/stratification programmes in near future clinical applications.

### Clinical data

Medical evaluation of IBD patients could not only serve for diagnosis or follow-up. Reuse and re-assessment of those data might be also helpful for making tailored and targeted therapy decisions in a cost-effective manner. For instance, taking advantage of the results from the tofacitinib OCTAVE Induction 1 and 2 clinical trials, Lees CW et al. could identify that baseline PMS, C-reactive protein (CRP) or cholesterol levels predict early clinical response [41]. Moreover, it has been reported that the nutritional status correlates with the response to VDZ, showing the non-responder patients an increased Malnutrition Universal Screening Tool (MUST) score at the initiation of therapy [42].

More sophisticated tools using clinical data are being developed. This is the case for the clinical decision support tool (CDST) for VDZ-treated patients, which integrates real-world data of five variables (no prior bowel surgery, no prior anti-TNF exposure, no prior fistulising disease, albumin and CRP levels), and it is able to discriminate the CD patients that will benefit the most from that therapy with high specificity since this CDST does not allow reliable predictions in anti-TNF-treated patients [43]. These results were corroborated by independent authors, who not only demonstrated that the CDST could predict remission in CD patients, but as observed for anti-TNF treated patients, that tool did not have predictor capacity on patients under UST therapy either [44], thereby confirming the specificity of this decision tool and its possible implementation in the routine clinical practice. Interestingly, a similar approach has been applied to UC patients with promising results in terms of clinical and endoscopic remission and specificity *versus* adalimumab [45].

Finally, applying new bioinformatic approaches, such as machine learning, provides new perspectives on the utility of conventional clinical parameters as non-invasive, easy-to-monitor and cheap predictive tools. Several studies are reviewing the applicability of common disease, biochemical or blood parameters and building novel predictive algorithms for anti-TNF or VDZ therapies with high performance [46–48]. Conventional predictive models used so far have the advantage of being simpler and more intuitive. Although this strategy had relative success, the use of artificial intelligence may help to put the pieces of the puzzle together of the overwhelming complexity of biologic and small molecules treatment resistances. However, this field is in an immature stage, and more studies using real-world data are necessary to guarantee the translation of bioinformatic data to useful prevention/therapeutic approaches for secondary care.

## Microbiome-derived data—in-depth analysis and outlook

Up to now, the participation of the intestinal microbiota in the physiopathology of IBD is out of the question, and it is considered one of the main factors responsible for intestinal inflammation development. New powerful sequencing techniques and bioinformatic analysis have allowed exponential growth in publications related to that topic, which revealed a prominent role for the resident commensal microorganisms in many of the mechanisms that lead to exacerbated intestinal inflammation. Therefore, it is not a surprise that the microbiome has been also postulated as a predictive tool for biologic therapy response (Table 2).

**Table 2:** Microbiota-derived predictive parameters and their association with the response to several biologic treatments. This table shows the utility of up-to-date metagenomic techniques in the designing of tailored therapeutic approaches for IBD patients

	FACTOR	LEVEL	PREDICTION	*Ref*
Microbiome-derived data	*Clostridiales*	Depletion	More severe conditions	49, 51, 52
	*Proteobacteria*	Enrichment		
	*Bifidobacterium*, *Clostridium colinum*, *Eubacterium rectale*, uncultured *Clostridiales* and *Vibrio*	High levels	R to anti-TNF therapy in paediatric IBD patients	53
	*Streptococcus mitis*	Low levels		
	*Clostridiales*	Low levels	NR to anti-TNF therapy	56,57
	*Roseburia inulinivorans* and *Burkholderiales*	High levels	R to VDZ therapy	58
	*Faecalibacterium*, *Escherichia* or *Shigella*	High levels	R to UST therapy	54

Dysbiosis and impaired bacterial metabolite production are commonly observed in IBD patients [49, 50]. In general, healthy controls present increased levels of alpha diversity, and amongst subjects with IBD, depletion of order *Clostridiales* and enrichment of *Proteobacteria* are associated with more severe conditions [49, 51, 52]. Interestingly, diversity and dysbiosis are recovered in responder patients to anti-cytokine therapy [49, 53, 54], and baseline microbial richness is related to later clinical remission in anti-TNF or UST-treated patients [55], which highlights the potential relationship between microbial composition and biologic agents efficacy. Indeed, recent studies have demonstrated the predictive value of particular bacterial genera at baseline in anti-TNF-treated patients. *Bifidobacterium*, *Clostridium colinum*, *Eubacterium rectale*, uncultured *Clostridiales* and *Vibrio* presented higher abundances in paediatric IBD patients responding to anti-TNF therapy. On the other hand, *Streptococcus mitis* showed decreased levels [53]. The individualised sensitivity and specificity analysis of all those bacterial types to predict anti-TNF response showed a prediction accuracy above 80%. Similarly, in adult IBD patients, a trained model using intestinal microbiota revealed a high accuracy in predicting IFX response, which is even further increased when used in combination with faecal calprotectin and Crohn’s disease activity index (CDAI) [49]. Noteworthy, the genus that contributed the most to the observed prognostic value is *Clostridiales* [49]. In fact, other studies also observed a reduction in Clostridia in non-responder patients to anti-TNF medication [56, 57], suggesting a strong association between this bacterial genus and the future outcome of that biologic therapy. That observed specificity was further supported by data obtained from VDZ-treated patients, in which therapy response correlated with increased abundances of *Roseburia inulinivorans* and different species of the *Burkholderiales* order at baseline [58], and the same occurred with Faecalibacterium, Escherichia, or Shigella genera in patients treated with UST [54]. Although the limited number of studies performed with all current IBD biologic therapies and the existence of discrepancies [59, 60] make not possible to apply these criteria for the stratification of patients at present, these data clearly pave the way for future interventions based on the quantification of specific bacterial genera/species before treatment for the discrimination of patients that most likely will achieve clinical remission with a given therapeutic option. Actually, a proof-of-concept study demonstrated that a microbiome signature composed of four bacterial markers displayed a high accuracy in discriminating responders from non-responders to anti-TNF therapy [61].

## Transcriptomic-derived data is boosting predictive and personalised approaches

Bioinformatics and artificial intelligence are no longer restricted to ultra-specialised research groups and centres. That technology is becoming available for most basic and clinical researchers, who are able to generate and integrate data that several years ago would not have been possible. At the same time, that increase in methodology independence and decrease in technical complexity will mean that any hospital would be capable of developing and implementing predictive algorithms/biomarkers without the participation of industrial partners, thereby shifting from reactive medicine to PPPM in a cost-effective manner and respecting the medical principle of justice since a directed genotyping and the application of a known algorithm would be possible with the already-existing resources of most health centres.

As discussed above, there have been identified several interesting biomarkers with high sensitivity and specificity for predicting the response to biologics. Although the isolation of a single-specific biomarker has great translational value because of its simplicity, the increased use of high-throughput technologies has revealed the enormous complexity of finding solid differences between responders and non-responders to the current IBD therapies (Table 3). Therefore, most likely, in the near future, the search for predictive factors will focus on big data and complex bioinformatic analysis. In that scenario, transcriptomics has emerged as an attractive tool, because of its ability to map and explore all expressed genes, a wide variety of analyses and the substantial amount of publicly available data. However, there is room for improvement in the integration and reproducibility of the results before applying this method in daily clinical practice.

**Table 3:** Transcriptomic-derived data is boosting predictive and personalised approaches. This table summarises the increasing number of transcriptomic studies and their relevance for achieving a predictive, preventive and personalised approach for IBD patients

	FACTOR	LEVEL	PREDICTION	*Ref*
Transcriptomic-derived data	SELE, AQP9, FPR2, TREM-1 and HCAR3	Decreased at baseline	R to IFX therapy	62
	IL7R signalling pathway	Increased in NR VDZ and IFX patients before treatment	NR to anti-TNF and VDZ therapy	63
	Seven-gene and a 13-gene signatures	Dysregulated at baseline	NR to anti-TNF therapy	65, 66
	Immune cells	Increased plasma cells at baseline	NR to anti-TNF therapy	27
		Increased neutrophils and activated CD4^+^ T cells before treatment	NR to VDZ or IFX therapy	68
	NFκB, TGF-β or JAK-STAT signalling pathway	Increased at baseline	NR to anti-TNF therapy	69, 70
	CRIP2, CXCL6, EMILIN1, GADD45B, LAMA4 and MAPKAPK2	Increased at baseline	NR to anti-TNF therapy	71
	IL23A, CXCL10, CCL5, CXCL5, CEBPB, LTB, SELE, CXCL9 and CCL24	Increased at baseline	NR to VDZ therapy	72

One of the main issues related to transcriptomic data is the lack of consistency for studies published. To overcome that problem, researchers commonly validate microarray or RNA-seq results in different cohorts/datasets. Following this workflow, several studies showed potential new biomarkers for biologic therapy success. For instance, the overlap of five datasets containing colon microarray data from responders and non-responders to IFX revealed five consistent differentiated expressed genes (DEGs), namely selectin E (SELE), aquaporin 9 (AQP9), formyl peptide receptor 2(FPR2), TREM-1 and hydroxycarboxylic acid receptor 3 (HCAR3) [62]. Functional validation of these findings would be necessary before using these biomarkers for the discrimination of IFX-responder IBD patients. Similarly, Belarif et al. demonstrated that the expression of the genes associated with the interleukin-7 receptor (IL7R) signalling pathway was altered before treatment in non-responder patients to IFX and VDZ [63], suggesting that IBD patients with altered IL7R expression would benefit more from the use of other biologics or small molecules. Also, this study demonstrated that IL7R blockade reduced intestinal inflammation in animal models and ex vivo human colon explant cultures, highlighting the potential of inhibiting this pathway in patients who are refractory for some of the currently approved therapies [64].

Apart from these single-specific biomarkers, transcriptomics makes it possible to isolate signatures composed of multiple genes. IBD is a complex disease, and those signatures may offer a complete vision of the putative predicting factors. Recently, it was reported seven-gene and 13-gene signatures capable of discriminating anti-TNF responders from non-responders with high accuracy in several mucosal biopsy datasets [65, 66]. In the same way, an even larger signature displayed an 87% accuracy [67], demonstrating the use of transcriptional groups could improve prediction effectiveness.

Transcriptome analysis also allows us to determine other parameters rather than gene expression. Estimation of immune cell composition or the dysregulated signalling pathways in colon tissue can be exploited for therapy response prediction. Very recently, it was found that the proportion of plasma cells at baseline could predict anti-TNF therapy response in two independent cohorts with an accuracy of 82% [27], and non-responders for either IFX or VDZ had increased abundance of neutrophils and activated CD4^+^ T cells before treatment [68]. Similarly, specific signalling pathways, namely NFκB, TGF-β or JAK-STAT, were enriched at baseline in non-responders IFX- or VDZ-treated patients [69, 70]. Altogether, these studies highlight the enormous potential and versatility of transcriptomic data for biomarker identification.

The abovementioned studies were mostly based on the analysis of public repositories of colon biopsies microarray data. However, in the last few years, other de novo experiments were performed using RNA-seq technology. These studies reported additional putative predictors for therapy response from new discovery cohorts. Particularly, Iacucci et al. identified six putative transcripts (CRIP2, CXCL6, EMILIN1, GADD45B, LAMA4 and MAPKAPK2) in colon tissue from discovery and validation cohorts with high sensitivity and specificity for anti-TNF therapy outcome prediction [71]. The same approach revealed another nine genes (IL23A, CXCL10, CCL5, CXCL5, CEBPB, LTB, SELE, CXCL9 and CCL24) that accurately discriminate VDZ treatment responders [72]. Also, as an attempt to identify biomarkers in peripheral blood, which would suppose less invasive monitoring of IBD patients, Mishra et al. and Salvador-Martín et al. reported a list of predictive transcripts for anti-TNF therapy in adult and paediatric patients [73, 74]. Unfortunately, RNA-seq data from patients treated with VDZ have not reported any DEG at baseline [75], which suggests that blood might not be ideal for searching biomarkers predicting VDZ response or more homogeneous discovery cohorts should be analysed since that study did not distinguish from CD and UC patients. Noteworthy, a very interesting study performed RNA-seq on sorted memory CD4 and T regulatory cells from peripheral blood mononuclear cells (PBMCs) and colon lamina propria lymphocytes of responder and non-responder patients to VDZ. Although the authors found several DEGs in samples from blood cells, most of the DEGs were obtained from the mucosal tissues [76], thereby highlighting the relevance of tissue and cell selection for biomarkers’ search and adding an extra level of complexity to this already-ambitious task.

## Conclusions and expert recommendations in the framework of predictive, preventive and personalised medicine

GIT is a unique system in the human body, which tolerates the luminal microbiota and protects involved tissues against harmful pathogenic flora and environmental invaders. Inflammatory bowel disease is a global health burden which carries lifelong morbidity affecting all age groups in populations with the disease-specific peak of the age groups ranging between 15 and 35 years. An accelerating incidence of IBD is reported for newly industrialised countries, whereas stabilising incidence but increasing prevalence is typical for countries with a Westernised lifestyle such as the European area and the USA. Although the aetiology of IBD is still largely unknown, the interplay between the genetic, environmental, immunological and microbial components is decisive for the disease manifestation, course, severity and individual outcomes. Contextually, the creation of an individualised patient profile is crucial for the cost-effective disease management in primary and secondary care. The proposed pathomechanisms include intestinal pathoflora and dysbiosis, chronic inflammation and mitochondrial impairments, amongst others, which collectively may reveal individual molecular signatures defining IBD subtypes and leading to clinical phenotypes, patient stratification and cost-effective protection against health-to-disease transition and treatments tailored to individualised patient profiles—all the pillars of an advanced 3PM approach. The paradigm change is proposed from reactive medical services to a predictive and personalised approach and targeted prevention and primary and secondary care of IBD meeting patient needs and benefiting the society at large. The below-listed innovative concepts are instrumental and strongly recommended for an effective paradigm change:

A. Very early onset IBD (VEOIBD) of monogenetic origin—paediatric gastroenterology

Although IBD may occur at any age, starting at birth till late elderly, the youngest IBD has a specific presentation, clinical course and aetiology. In contrast to the adult-onset IBD, usually representing a complex disease with synergies between the host genetic susceptibility and environmental triggers [77], VEOIBD patients diagnosed before 6 years of age demonstrate specific IBD-subtype including increased incidence of a monogenetic disease [78]. The monogenic categories identified so far are hyperinflammatory and autoinflammatory disorders, epithelial cell and adaptive immune defects as well as innate immune/bacterial clearance and recognition defects, which represent well-distinguishable IBD subtypes and demand individualised treatment algorithms.

B. Pathobionts as diagnostic and treatment target

Besides the concept of dysbiosis (loss of beneficial microbial species), which per evidence can exacerbate IBD, recently collected research data identified the so-called pathobionts, intestinal microflora of bacterial and fungal origin with pathogenic qualities highly relevant for the IBD onset. Pathobionts are considered a potent target for the relevant IBD subtype [79].

C. Impaired wound healing associated with specific IBD subtypes

Non-healing wounds are another potent biomarker for a specific IBD subtype, e.g. in patients with CD demonstrating *Debaryomyces hansenii* under pathobionts [79]. Since specifically neutrophils are associated with pro-inflammatory mechanisms which cause non-healing wounds and intestinal inflammation, several research groups hypothesised that restoration of neutrophil function and normalisation of neutrophil apoptosis may lead to improved wound healing and mitigation of the IBD symptoms [80, 81].

D. Stress-related IBD-subtypes—relevance for primary and secondary care

Stress of different origins is known to influence the brain-gut axis functionality contributing therefore to the development and severity of gastrointestinal disorders, including IBD. Various stressors such as psychological distress and exposure to high and low temperatures (heat and cold stress provocation) may significantly worsen the disease course in IBD patients [82]. Contextually, in primary care, specialised screening programmes are of great clinical relevance in identifying vulnerable individuals with increased stress sensitivity such as Flammer syndrome phenotype carriers [83–86].

E. Compromised mitochondrial health and decreased mitophagy as a potent treatment target in primary and secondary care

Compromised mitochondrial health is the key to the pathophysiology of chronic inflammatory diseases, ranging from cancer to neurodegenerative disorders and involving the gut–brain axis [87, 88].

To this end, the gut deals with high concentrations of bacteria and a spectrum of their metabolites, immune-active and damage-associated substances, xenobiotics and environmental toxins which synergistically may damage the mitochondria and disturb mitochondrial homeostasis well measurable, e.g. in case of suppressed mitophagy—an indicator of a protective response induced by mitochondrial-derived reactive oxygen species (ROS) during intestinal inflammation [89].

Indeed, mitochondrial dynamics in cultured gut epithelial lines become compromised in the presence of co-cultured pathobionts [90]—see point B. Further, an excess of damaged mitochondria within gut enterocytes was demonstrated to induce colitis in corresponding experimental sets [91, 92].

Overall, 3PM innovation is challenging and requires the involvement of multi-professional expertise [93].

## Data Availability

Not applicable.

## Abbreviations

*ADA*: Adalimumab
*AQP9*: Aquaporin 9
*CD*: Crohn’s disease
*CDAI*: Crohn’s disease activity index
*CDST*: Clinical decision support tool
*CRP*: C-reactive protein
*DEG*: Differentiated expressed gene
*FPR2*: Formyl peptide receptor 2
*GIT*: Gastrointestinal tract
*HCAR3*: Hydroxycarboxylic acid receptor 3
*IBD*: Inflammatory bowel disease
*IFX*: Infliximab
*IL7R*: Interleukin-7 receptor
*LI*: Lichtiger index
*MES*: Mayo endoscopic subscore
*MUST*: Malnutrition Universal Screening Tool
*NAMPT*: Nicotinamide phosphoribosyltransferase
*NSAID*: Nonsteroidal anti-inflammatory drug
*OSM*: Oncostatin M
*PBMC*: Peripheral blood mononuclear cell
*PMS*: Partial Mayo score
*PPPM/3PM*: Predictive preventive personalised medicine
*ROS*: Reactive oxygen species
*SELE*: Selectin E
*TNF*: Tumour necrosis factor
*TREM-1*: Triggering receptor expressed on myeloid cells 1
*UC*: Ulcerative colitis
*UST*: Ustekinumab
*VSZ*: Vedolizumab
*VEOIBD*: Very-early-onset IBD
